# Bis(dihydrogen norfloxacinium) tri-μ_2_-chlorido-bis­[trichloridobismuthate(III)] chloride dihydrate

**DOI:** 10.1107/S1600536808001244

**Published:** 2008-01-23

**Authors:** A. V. Gerasimenko, E. T. Karaseva, A. V. Polishchuk

**Affiliations:** a Institute of Chemistry, FEB RAS, Prospekt 100-letiya Vladivostoka 159, Vladivostok 690022, Russian Federation

## Abstract

The title compound, {systematic name: (3-carb­oxy-1-ethyl-6-fluoro-7-piperazin-4-ium-1-yl-1*H*-quinolin-4-yl­idene)oxonium tri-μ_2_-chlorido-bis­[trichloridobismuthate(III)] chloride dihydrate], (C_16_H_20_FN_3_O_3_)_2_[Bi_2_Cl_9_]Cl·2H_2_O, is composed of [Bi_2_Cl_9_]^3−^ anions lying on crystallographic twofold rotation axes, Cl^−^ anions also on twofold axes, C_16_H_20_FN_3_O_3_^2+^ cations, and water mol­ecules. The Bi^III^ coordination polyhedron is a distorted octa­hedron and two such octa­hedra share a triangular face to form the complex anion. There are three short terminal Bi—Cl bonds [2.5471 (6)–2.5781(5 Å] and three longer bridging bonds [2.8599 (5)–2.9984 (6) Å] in each octa­hedron. Anions, cations and water mol­ecules are linked by hydrogen bonds to form a three-dimensional network. There are also π–π stacking inter­actions between quinoline ring systems, with an inter­planar distance of 3.27 (1) Å.

## Related literature

For the Cambridge Structural Database (Version 5.28) used to identify related structures, see: Allen (2002 ▶).
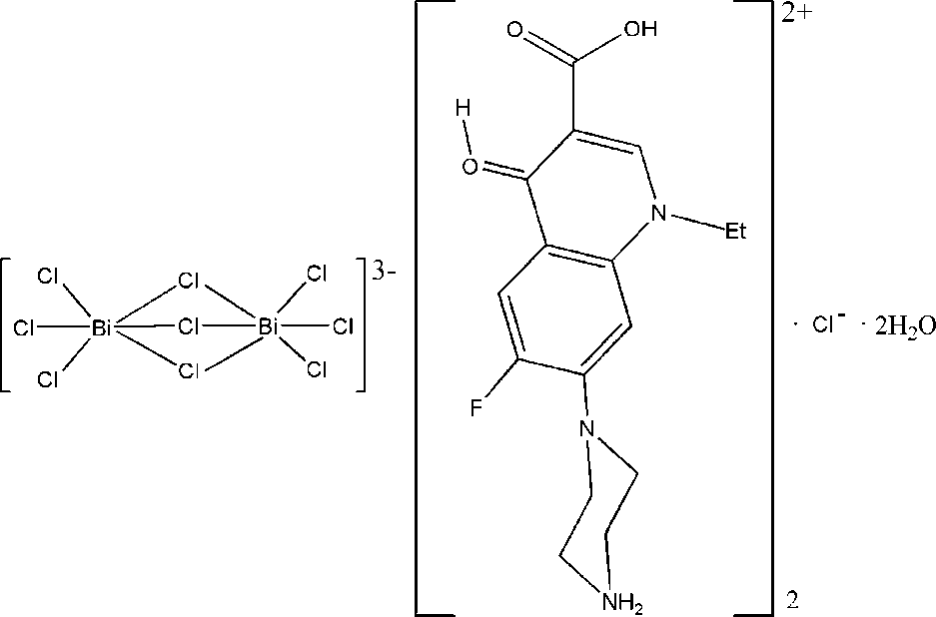


## Experimental

### 

#### Crystal data

(C_16_H_20_FN_3_O_3_)_2_[Bi_2_Cl_9_]Cl·2H_2_O
*M* *_r_* = 1451.19
Monoclinic, 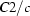

*a* = 13.9109 (12) Å
*b* = 22.7104 (19) Å
*c* = 14.5964 (12) Å
β = 92.798 (2)°
*V* = 4605.8 (7) Å^3^
*Z* = 4
Mo *K*α radiation
μ = 8.27 mm^−1^
*T* = 203 (2) K
0.27 × 0.22 × 0.17 mm

#### Data collection

Bruker SMART 1000 CCD area-detector diffractometer
Absorption correction: Gaussian (*SADABS*; Bruker, 2003 ▶) *T*_min_ = 0.180, *T*_max_ = 0.334
16736 measured reflections
6987 independent reflections
6081 reflections with *I* > 2σ(*I*)
*R*_int_ = 0.031

#### Refinement

*R*[*F* ^2^ > 2σ(*F* ^2^)] = 0.025
*wR*(*F* ^2^) = 0.061
*S* = 1.06
6987 reflections
282 parameters
1 restraint
H atoms treated by a mixture of independent and constrained refinement
Δρ_max_ = 1.58 e Å^−3^
Δρ_min_ = −0.84 e Å^−3^

### 

Data collection: *SMART* (Bruker, 1998 ▶); cell refinement: *SAINT* (Bruker, 2003 ▶); data reduction: *SAINT*; program(s) used to solve structure: *SHELXTL* (Sheldrick, 2008 ▶); program(s) used to refine structure: *SHELXTL*; molecular graphics: *XP* in *SHELXTL*; software used to prepare material for publication: *publCIF* (Version 1.9.0; Westrip, 2008 ▶).

## Supplementary Material

Crystal structure: contains datablocks I, global. DOI: 10.1107/S1600536808001244/cf2178sup1.cif

Structure factors: contains datablocks I. DOI: 10.1107/S1600536808001244/cf2178Isup2.hkl

Additional supplementary materials:  crystallographic information; 3D view; checkCIF report

## Figures and Tables

**Table 1:** Selected bond lengths (Å)

Bi1⋯Bi1^i^	3.7851 (3)
Bi1—Cl4	2.5471 (6)
Bi1—Cl3	2.5497 (5)
Bi1—Cl5	2.5781 (5)
Bi1—Cl1	2.8599 (5)
Bi1—Cl2	2.9194 (6)
Bi1—Cl2^i^	2.9984 (6)

Symmetry code: (i) 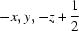.

**Table 2:** Hydrogen-bond geometry (Å, °)

*D*—H⋯*A*	*D*—H	H⋯*A*	*D*⋯*A*	*D*—H⋯*A*
O3—H3⋯O1	0.83	1.85	2.583 (2)	146
O2—H2⋯Cl6^ii^	0.83	2.19	3.0150 (15)	173
O4—H4*A*⋯Cl5	0.715 (18)	2.563 (18)	3.270 (2)	170 (4)
O4—H4*B*⋯Cl6	0.717 (19)	2.47 (2)	3.146 (2)	157 (4)
N3—H3*A*⋯Cl2^iii^	0.91	2.51	3.3961 (19)	165
N3—H3*B*⋯O4^iv^	0.91	1.82	2.693 (3)	160

Symmetry codes: (ii) 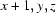; (iii) 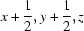; (iv) 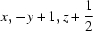.

**Fig. 1.**
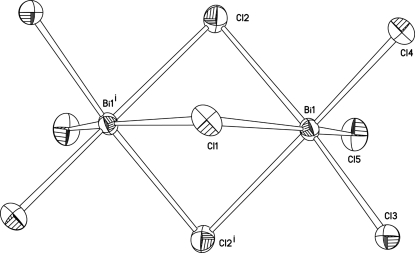
A view of the dinuclear [Bi2Cl9]3- complex, with displacement ellipsoids drawn at the 50% probability level. [Symmetry code: (i) -x, y, 1/2 - z.]

**Fig. 2.**
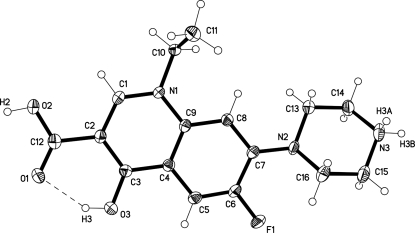
A view of the nfH32+ canion, with displacement ellipsoids drawn at the 50% probability level. The intramolecular hydrogen bond is shown as a dashed line.

**Fig. 3.**
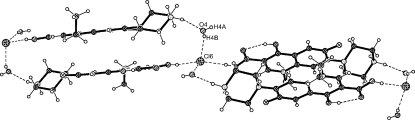
Fragment of the zigzag chain formed from water molecules, chloride anions and nfH32+ canions, with hydrogen bonds shown as dashed lines.

**Fig. 4.**
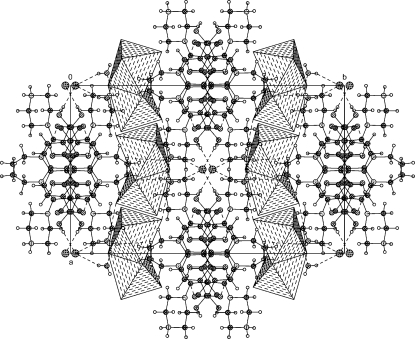
The crystal structure of the title compound viewed along the c axis. Dashed lines represent hydrogen bonds.

## supplementary crystallographic information

### Comment

Norfloxacin (*nfH*) belongs to the second-generation quinolone antimicrobial agents. According to a search of the Cambridge Structural Database (CSD, Version 5.28; Allen, 2002), well determined relevant structures are those where norfloxacin acts as an anion, a singly protonated cation or a zwitterion. The present research deals with the synthesis and structure of a chlorido-bismuth complex with the doubly protonated cation of norfloxacin (*nfH_3_*)^2+^.

The asymmetric unit of the title compound, (I), contains one Bi atom, five chlorine atoms, one *nfH_3_* cation and one H_2_O molecule. The Bi atoms are coordinated by six Cl atoms in a distorted octahedral geometry. Two Bi-centred octahedra are linked by triple Cl bridges to form a dinuclear [Bi_2_Cl_9_]^3-^ complex (Fig. 1), which lies on a twofold rotation axis, with a Bi···Bi distance of 3.7851 (3) Å. In the Bi-centred octahedra there are three short terminal Bi—Cl bonds [2.5471 (6)–2.5781 (5) Å] and three longer bridging bonds [2.8599 (5)–2.9984(6] Å). These anions pack in columns parallel to the [101] direction.

The protonation of *nfH_3_*^2+^ is realised on the carbonyl atom O3 and N3 of the piperazine ring (Fig. 2). The hydrogen atom H3 is linked by an intramolecular hydrogen bond with O1 of the carboxyl group. O2 and N3 in the cation act as hydrogen-bond donors, *via* H2, H3A and H3B.

Water molecules, uncoordinated chloride anions (Cl6) and *nfH_3_*^2+^ cations are linked in zigzag chains by hydrogen bonds parallel to the [102] direction (Fig. 3). In the chain, the *nfH_3_*^2+^ cations are pairwise parallel (as a result of inversion symmetry), and there exist also π–π interactions between quinoline ring systems, with an interplanar distance of 3.27 (1) Å.

The combination of the hydrogen bonds and π–π stacking generates a three-dimensional network (Fig. 4).

### Experimental

Bi(OH)_3_ (0.052 g, 0.2 mmol) was reacted with *nfH* (0.066 g, 0.2 mmol) in an aqueous solution of HCl (21%, 20 ml). Yellow crystals were obtained after evaporation for 72 h at room temperature.

### Refinement

H atoms (for H_2_O) were located in a difference map and refined with *U*_iso_(H) = 1.5*U*_eq_(O) and the O—H distances were restrained to be similar. The other H atoms were positioned with idealized geometry using a riding model with C—H = 0.94, 0.97 and 0.98 Å; N—H = 0.91 Å and O—H = 0.83 Å. All H atoms were refined with *U*_iso_ set to 1.2 or 1.5 times *U*_eq_ of the parent atom. The maximum peak and the deepest hole are located 0.77 Å and 1.33 Å from Bi, respectively.

### Crystal data

**Table:**

(C_16_H_20_FN_3_O_3_)_2_[Bi_2_Cl_9_]Cl·2H_2_O	*F*_000_ = 2784
*M**_r_* = 1451.19	*D*_x_ = 2.093 Mg m^−^^3^
Monoclinic, *C*2/*c*	Mo *K*α radiation λ = 0.71073 Å
*a* = 13.9109 (12) Å	Cell parameters from 951 reflections
*b* = 22.7104 (19) Å	θ = 3.9–30.6º
*c* = 14.5964 (12) Å	µ = 8.27 mm^−^^1^
β = 92.798 (2)º	*T* = 203 (2) K
*V* = 4605.8 (7) Å^3^	Prism, yellow
*Z* = 4	0.27 × 0.22 × 0.17 mm

### Data collection

**Table:**

Bruker SMART 1000 CCD area-detector diffractometer	6987 independent reflections
Radiation source: fine-focus sealed tube	6081 reflections with *I* > 2σ(*I*)
Monochromator: graphite	*R*_int_ = 0.031
Detector resolution: 8.33 pixels mm^-1^	θ_max_ = 31.5º
*T* = 203(2) K	θ_min_ = 3.6º
φ and ω scans	*h* = −20→15
Absorption correction: Gaussian(SADABS; Bruker, 2003)	*k* = −31→32
*T*_min_ = 0.180, *T*_max_ = 0.334	*l* = −20→14
16736 measured reflections	

### Refinement

**Table:**

Refinement on *F*^2^	Secondary atom site location: difference Fourier map
Least-squares matrix: full	Hydrogen site location: inferred from neighbouring sites
*R*[*F*^2^ > 2σ(*F*^2^)] = 0.025	H atoms treated by a mixture of independent and constrained refinement
*wR*(*F*^2^) = 0.061	*w* = 1/[σ^2^(*F*_o_^2^) + (0.0269*P*)^2^ + 1.9407*P*] where *P* = (*F*_o_^2^ + 2*F*_c_^2^)/3
*S* = 1.06	(Δ/σ)_max_ = 0.012
6987 reflections	Δρ_max_ = 1.58 e Å^−^^3^
282 parameters	Δρ_min_ = −0.84 e Å^−^^3^
1 restraint	Extinction correction: SHELXL97 (Sheldrick, 2008), Fc^*^=kFc[1+0.001xFc^2^λ^3^/sin(2θ)]^-1/4^
Primary atom site location: structure-invariant direct methods	Extinction coefficient: 0.000362 (17)

### Special details

**Table:**

Geometry. All e.s.d.'s (except the e.s.d. in the dihedral angle between two l.s. planes) are estimated using the full covariance matrix. The cell e.s.d.'s are taken into account individually in the estimation of e.s.d.'s in distances, angles and torsion angles; correlations between e.s.d.'s in cell parameters are only used when they are defined by crystal symmetry. An approximate (isotropic) treatment of cell e.s.d.'s is used for estimating e.s.d.'s involving l.s. planes.
Refinement. Refinement of *F*^2^ against ALL reflections. The weighted *R*-factor *wR* and goodness of fit *S* are based on *F*^2^, conventional *R*-factors *R* are based on *F*, with *F* set to zero for negative *F*^2^. The threshold expression of *F*^2^ > σ(*F*^2^) is used only for calculating *R*-factors(gt) *etc*. and is not relevant to the choice of reflections for refinement. *R*-factors based on *F*^2^ are statistically about twice as large as those based on *F*, and *R*- factors based on ALL data will be even larger.

### Fractional atomic coordinates and isotropic or equivalent isotropic displacement parameters (Å^2^)

**Table:**

	*x*	*y*	*z*	*U*_iso_*/*U*_eq_	
Bi1	0.125399 (5)	0.231497 (3)	0.205209 (5)	0.01711 (2)	
Cl1	0.0000	0.13708 (3)	0.2500	0.02433 (15)	
Cl2	0.06458 (4)	0.26380 (2)	0.38711 (4)	0.02637 (11)	
Cl3	0.16112 (4)	0.18802 (2)	0.04945 (3)	0.02670 (11)	
Cl4	0.27484 (4)	0.18468 (3)	0.28222 (4)	0.03193 (13)	
Cl5	0.20122 (4)	0.33350 (2)	0.18044 (4)	0.03441 (13)	
Cl6	0.5000	0.48180 (3)	0.2500	0.0356 (2)	
F1	0.75259 (8)	0.46603 (5)	0.43340 (9)	0.0275 (3)	
O1	1.25735 (10)	0.45323 (6)	0.28600 (10)	0.0262 (3)	
O2	1.32428 (11)	0.54074 (6)	0.32107 (12)	0.0317 (4)	
H2	1.3714	0.5259	0.2970	0.048*	
O3	1.08058 (10)	0.43447 (6)	0.32403 (10)	0.0225 (3)	
H3	1.1348	0.4259	0.3067	0.034*	
O4	0.42564 (14)	0.36716 (10)	0.14845 (15)	0.0632 (6)	
H4A	0.3777 (12)	0.3561 (14)	0.152 (3)	0.076*	
H4B	0.429 (3)	0.3968 (8)	0.165 (2)	0.076*	
N1	1.07419 (11)	0.60588 (7)	0.41452 (11)	0.0167 (3)	
N2	0.73624 (11)	0.58287 (7)	0.48464 (11)	0.0200 (4)	
N3	0.56019 (12)	0.64241 (8)	0.52271 (13)	0.0275 (4)	
H3A	0.5558	0.6702	0.4778	0.033*	
H3B	0.5084	0.6464	0.5579	0.033*	
C1	1.15439 (13)	0.58404 (8)	0.38258 (13)	0.0184 (4)	
H1	1.2091	0.6083	0.3820	0.022*	
C2	1.16090 (13)	0.52665 (8)	0.34983 (13)	0.0179 (4)	
C3	1.08010 (13)	0.48980 (8)	0.35148 (13)	0.0172 (4)	
C4	0.99316 (13)	0.51308 (8)	0.38424 (13)	0.0167 (4)	
C5	0.91002 (14)	0.47809 (8)	0.38934 (13)	0.0192 (4)	
H5	0.9105	0.4386	0.3700	0.023*	
C6	0.82924 (13)	0.50185 (8)	0.42245 (13)	0.0193 (4)	
C7	0.82172 (13)	0.56183 (8)	0.45095 (13)	0.0180 (4)	
C8	0.90503 (13)	0.59551 (8)	0.44819 (13)	0.0176 (4)	
H8	0.9043	0.6348	0.4684	0.021*	
C9	0.99058 (13)	0.57178 (8)	0.41560 (13)	0.0170 (4)	
C10	1.07597 (14)	0.66786 (8)	0.44871 (14)	0.0211 (4)	
H10A	1.1427	0.6794	0.4639	0.025*	
H10B	1.0406	0.6701	0.5050	0.025*	
C11	1.03232 (16)	0.71041 (9)	0.37950 (16)	0.0282 (5)	
H11A	1.0642	0.7065	0.3222	0.042*	
H11B	1.0403	0.7503	0.4023	0.042*	
H11C	0.9643	0.7019	0.3694	0.042*	
C12	1.25206 (14)	0.50349 (9)	0.31606 (14)	0.0213 (4)	
C13	0.73860 (14)	0.64186 (9)	0.52505 (14)	0.0228 (4)	
H13A	0.7405	0.6714	0.4763	0.027*	
H13B	0.7969	0.6463	0.5650	0.027*	
C14	0.65017 (15)	0.65186 (10)	0.58046 (16)	0.0270 (5)	
H14A	0.6516	0.6247	0.6327	0.032*	
H14B	0.6511	0.6921	0.6045	0.032*	
C15	0.55900 (15)	0.58264 (10)	0.48029 (16)	0.0284 (5)	
H15A	0.5012	0.5782	0.4397	0.034*	
H15B	0.5572	0.5526	0.5284	0.034*	
C16	0.64805 (14)	0.57405 (10)	0.42577 (14)	0.0254 (5)	
H16A	0.6480	0.5342	0.4001	0.031*	
H16B	0.6471	0.6021	0.3748	0.031*	

### Atomic displacement parameters (Å^2^)

**Table:**

	*U*^11^	*U*^22^	*U*^33^	*U*^12^	*U*^13^	*U*^23^
Bi1	0.01352 (3)	0.01870 (3)	0.01912 (3)	−0.00108 (2)	0.00093 (2)	−0.00093 (2)
Cl1	0.0228 (3)	0.0167 (3)	0.0330 (4)	0.000	−0.0039 (3)	0.000
Cl2	0.0241 (2)	0.0284 (2)	0.0269 (2)	−0.00434 (18)	0.00425 (19)	−0.00849 (19)
Cl3	0.0214 (2)	0.0372 (2)	0.0214 (2)	0.00634 (19)	−0.00045 (18)	−0.0049 (2)
Cl4	0.0214 (2)	0.0466 (3)	0.0275 (3)	0.0063 (2)	−0.00225 (19)	0.0042 (2)
Cl5	0.0332 (3)	0.0226 (2)	0.0480 (3)	−0.0083 (2)	0.0071 (2)	−0.0018 (2)
Cl6	0.0209 (3)	0.0200 (3)	0.0671 (5)	0.000	0.0154 (3)	0.000
F1	0.0198 (5)	0.0281 (6)	0.0351 (7)	−0.0095 (5)	0.0049 (5)	−0.0042 (5)
O1	0.0213 (7)	0.0252 (7)	0.0324 (8)	0.0038 (6)	0.0036 (6)	−0.0085 (6)
O2	0.0182 (7)	0.0258 (7)	0.0523 (10)	−0.0008 (6)	0.0126 (6)	−0.0086 (7)
O3	0.0206 (6)	0.0201 (6)	0.0267 (7)	0.0011 (5)	0.0017 (6)	−0.0056 (5)
O4	0.0324 (9)	0.0866 (13)	0.0726 (13)	−0.0253 (9)	0.0216 (9)	−0.0498 (11)
N1	0.0134 (6)	0.0167 (6)	0.0199 (7)	−0.0018 (5)	0.0008 (6)	−0.0012 (6)
N2	0.0109 (7)	0.0268 (7)	0.0223 (8)	0.0003 (6)	−0.0003 (6)	−0.0025 (7)
N3	0.0158 (7)	0.0310 (8)	0.0360 (10)	0.0057 (7)	0.0045 (7)	0.0098 (8)
C1	0.0142 (8)	0.0213 (8)	0.0196 (9)	−0.0020 (6)	0.0008 (7)	0.0016 (7)
C2	0.0151 (8)	0.0208 (8)	0.0178 (8)	0.0006 (7)	0.0013 (7)	−0.0004 (7)
C3	0.0183 (8)	0.0163 (7)	0.0167 (8)	0.0006 (6)	−0.0004 (7)	0.0005 (7)
C4	0.0157 (8)	0.0186 (8)	0.0159 (8)	−0.0006 (6)	−0.0002 (6)	−0.0002 (7)
C5	0.0194 (8)	0.0209 (8)	0.0171 (8)	−0.0032 (7)	−0.0002 (7)	−0.0016 (7)
C6	0.0148 (8)	0.0240 (8)	0.0189 (9)	−0.0045 (7)	−0.0002 (7)	−0.0007 (7)
C7	0.0159 (8)	0.0211 (8)	0.0168 (8)	−0.0015 (7)	−0.0016 (7)	0.0003 (7)
C8	0.0165 (8)	0.0183 (8)	0.0180 (8)	0.0006 (6)	0.0001 (7)	−0.0016 (7)
C9	0.0141 (8)	0.0200 (8)	0.0168 (8)	−0.0002 (6)	−0.0004 (6)	0.0006 (7)
C10	0.0171 (8)	0.0192 (8)	0.0273 (10)	−0.0027 (7)	0.0027 (7)	−0.0087 (7)
C11	0.0277 (10)	0.0224 (9)	0.0348 (12)	0.0019 (8)	0.0045 (9)	0.0004 (9)
C12	0.0200 (9)	0.0248 (9)	0.0191 (9)	0.0019 (7)	0.0008 (7)	0.0007 (7)
C13	0.0175 (8)	0.0232 (9)	0.0277 (10)	0.0006 (7)	0.0029 (7)	−0.0024 (8)
C14	0.0199 (9)	0.0276 (9)	0.0338 (11)	0.0043 (8)	0.0048 (8)	0.0006 (9)
C15	0.0157 (9)	0.0325 (10)	0.0367 (12)	0.0009 (8)	−0.0014 (8)	0.0053 (9)
C16	0.0169 (9)	0.0361 (10)	0.0229 (10)	−0.0016 (8)	−0.0028 (7)	0.0020 (8)

### Geometric parameters (Å, °)

**Table:**

Bi1—Bi1^i^	3.7851 (3)	C1—H1	0.940
Bi1—Cl4	2.5471 (6)	C2—C3	1.402 (3)
Bi1—Cl3	2.5497 (5)	C2—C12	1.479 (3)
Bi1—Cl5	2.5781 (5)	C3—C4	1.424 (3)
Bi1—Cl1	2.8599 (5)	C4—C5	1.408 (3)
Bi1—Cl2	2.9194 (6)	C4—C9	1.411 (3)
Bi1—Cl2^i^	2.9984 (6)	C5—C6	1.357 (3)
Cl1—Bi1^i^	2.8599 (5)	C5—H5	0.940
Cl2—Bi1^i^	2.9984 (6)	C6—C7	1.430 (3)
F1—C6	1.357 (2)	C7—C8	1.391 (3)
O1—C12	1.226 (2)	C8—C9	1.410 (3)
O2—C12	1.313 (2)	C8—H8	0.940
O2—H2	0.830	C10—C11	1.504 (3)
O3—C3	1.319 (2)	C10—H10A	0.980
O3—H3	0.830	C10—H10B	0.980
O4—H4A	0.715 (18)	C11—H11A	0.970
O4—H4B	0.717 (19)	C11—H11B	0.970
N1—C1	1.326 (2)	C11—H11C	0.970
N1—C9	1.398 (2)	C13—C14	1.522 (3)
N1—C10	1.493 (2)	C13—H13A	0.980
N2—C7	1.393 (2)	C13—H13B	0.980
N2—C13	1.464 (3)	C14—H14A	0.980
N2—C16	1.477 (3)	C14—H14B	0.980
N3—C14	1.490 (3)	C15—C16	1.517 (3)
N3—C15	1.492 (3)	C15—H15A	0.980
N3—H3A	0.910	C15—H15B	0.980
N3—H3B	0.910	C16—H16A	0.980
C1—C2	1.393 (3)	C16—H16B	0.980
			
Cl4—Bi1—Cl3	92.375 (18)	F1—C6—C7	117.90 (16)
Cl4—Bi1—Cl5	96.10 (2)	C8—C7—N2	123.26 (17)
Cl3—Bi1—Cl5	97.124 (19)	C8—C7—C6	116.27 (17)
Cl4—Bi1—Cl1	94.585 (16)	N2—C7—C6	120.35 (16)
Cl3—Bi1—Cl1	93.506 (14)	C7—C8—C9	121.19 (17)
Cl5—Bi1—Cl1	164.578 (16)	C7—C8—H8	119.4
Cl4—Bi1—Cl2	88.285 (17)	C9—C8—H8	119.4
Cl3—Bi1—Cl2	170.408 (16)	N1—C9—C8	120.56 (16)
Cl5—Bi1—Cl2	92.318 (17)	N1—C9—C4	119.04 (16)
Cl1—Bi1—Cl2	76.903 (12)	C8—C9—C4	120.39 (16)
Cl4—Bi1—Cl2^i^	169.401 (17)	N1—C10—C11	112.43 (16)
Cl3—Bi1—Cl2^i^	84.255 (16)	N1—C10—H10A	109.1
Cl5—Bi1—Cl2^i^	94.291 (17)	C11—C10—H10A	109.1
Cl1—Bi1—Cl2^i^	75.647 (12)	N1—C10—H10B	109.1
Cl2—Bi1—Cl2^i^	93.385 (16)	C11—C10—H10B	109.1
Bi1—Cl1—Bi1^i^	82.867 (18)	H10A—C10—H10B	107.9
Bi1—Cl2—Bi1^i^	79.513 (14)	C10—C11—H11A	109.5
C12—O2—H2	109.5	C10—C11—H11B	109.5
C3—O3—H3	109.5	H11A—C11—H11B	109.5
H4A—O4—H4B	110 (4)	C10—C11—H11C	109.5
C1—N1—C9	120.76 (15)	H11A—C11—H11C	109.5
C1—N1—C10	118.00 (15)	H11B—C11—H11C	109.5
C9—N1—C10	121.24 (15)	O1—C12—O2	124.09 (19)
C7—N2—C13	116.96 (15)	O1—C12—C2	121.17 (18)
C7—N2—C16	116.57 (16)	O2—C12—C2	114.74 (17)
C13—N2—C16	111.13 (16)	N2—C13—C14	110.36 (16)
C14—N3—C15	110.96 (16)	N2—C13—H13A	109.6
C14—N3—H3A	109.4	C14—C13—H13A	109.6
C15—N3—H3A	109.4	N2—C13—H13B	109.6
C14—N3—H3B	109.4	C14—C13—H13B	109.6
C15—N3—H3B	109.4	H13A—C13—H13B	108.1
H3A—N3—H3B	108.0	N3—C14—C13	110.88 (18)
N1—C1—C2	122.72 (17)	N3—C14—H14A	109.5
N1—C1—H1	118.6	C13—C14—H14A	109.5
C2—C1—H1	118.6	N3—C14—H14B	109.5
C1—C2—C3	119.14 (17)	C13—C14—H14B	109.5
C1—C2—C12	121.21 (17)	H14A—C14—H14B	108.1
C3—C2—C12	119.62 (16)	N3—C15—C16	110.00 (17)
O3—C3—C2	123.19 (17)	N3—C15—H15A	109.7
O3—C3—C4	118.21 (16)	C16—C15—H15A	109.7
C2—C3—C4	118.61 (16)	N3—C15—H15B	109.7
C5—C4—C9	118.83 (17)	C16—C15—H15B	109.7
C5—C4—C3	121.43 (17)	H15A—C15—H15B	108.2
C9—C4—C3	119.70 (16)	N2—C16—C15	110.71 (17)
C6—C5—C4	119.41 (17)	N2—C16—H16A	109.5
C6—C5—H5	120.3	C15—C16—H16A	109.5
C4—C5—H5	120.3	N2—C16—H16B	109.5
C5—C6—F1	118.21 (17)	C15—C16—H16B	109.5
C5—C6—C7	123.80 (17)	H16A—C16—H16B	108.1

Symmetry codes: (i) −*x*, *y*, −*z*+1/2.

### Hydrogen-bond geometry (Å, °)

**Table:**

*D*—H···*A*	*D*—H	H···*A*	*D*···*A*	*D*—H···*A*
O3—H3···O1	0.83	1.85	2.583 (2)	146
O2—H2···Cl6^ii^	0.83	2.19	3.0150 (15)	173
O4—H4A···Cl5	0.715 (18)	2.563 (18)	3.270 (2)	170 (4)
O4—H4B···Cl6	0.717 (19)	2.47 (2)	3.146 (2)	157 (4)
N3—H3A···Cl2^iii^	0.91	2.51	3.3961 (19)	165
N3—H3B···O4^iv^	0.91	1.82	2.693 (3)	160

Symmetry codes: (ii) *x*+1, *y*, *z*; (iii) *x*+1/2, *y*+1/2, *z*; (iv) *x*, −*y*+1, *z*+1/2.
